# Shearwater Foraging in the Southern Ocean: The Roles of Prey Availability and Winds

**DOI:** 10.1371/journal.pone.0010960

**Published:** 2010-06-04

**Authors:** Ben Raymond, Scott A. Shaffer, Serguei Sokolov, Eric J. Woehler, Daniel P. Costa, Luke Einoder, Mark Hindell, Graham Hosie, Matt Pinkerton, Paul M. Sagar, Darren Scott, Adam Smith, David R. Thompson, Caitlin Vertigan, Henri Weimerskirch

**Affiliations:** 1 Australian Antarctic Division, Kingston, Tasmania, Australia; 2 Department of Biological Sciences, San Jose State University, San Jose, California, United States of America; 3 CSIRO Wealth from Oceans National Research Flagship, CSIRO Marine Research and Antarctic Climate and Ecosystems Cooperative Research Centre, Hobart, Tasmania, Australia; 4 School of Zoology, University of Tasmania, Hobart, Tasmania, Australia; 5 Ecology and Evolutionary Biology, University of California, Santa Cruz, California, United States of America; 6 National Institute of Water and Atmospheric Research Ltd., Taihoro Nukurangi, Kilbirnie, Wellington, New Zealand; 7 National Institute of Water and Atmospheric Research Ltd., Taihoro Nukurangi, Christchurch, New Zealand; 8 Department of Zoology, University of Otago, Dunedin, New Zealand; 9 Institute of Information and Mathematical Sciences, Massey University, Albany, Auckland, New Zealand; 10 Centre d'Etudes Biologiques de Chizé, Centre National de la Recherche Scientifique, Villiers en Bois, France; Institut Pluridisciplinaire Hubert Curien, France

## Abstract

**Background:**

Sooty (*Puffinus griseus*) and short-tailed (*P. tenuirostris*) shearwaters are abundant seabirds that range widely across global oceans. Understanding the foraging ecology of these species in the Southern Ocean is important for monitoring and ecosystem conservation and management.

**Methodology/Principal Findings:**

Tracking data from sooty and short-tailed shearwaters from three regions of New Zealand and Australia were combined with at-sea observations of shearwaters in the Southern Ocean, physical oceanography, near-surface copepod distributions, pelagic trawl data, and synoptic near-surface winds. Shearwaters from all three regions foraged in the Polar Front zone, and showed particular overlap in the region around 140°E. Short-tailed shearwaters from South Australia also foraged in Antarctic waters south of the Polar Front. The spatial distribution of shearwater foraging effort in the Polar Front zone was matched by patterns in large-scale upwelling, primary production, and abundances of copepods and myctophid fish. Oceanic winds were found to be broad determinants of foraging distribution, and of the flight paths taken by the birds on long foraging trips to Antarctic waters.

**Conclusions/Significance:**

The shearwaters displayed foraging site fidelity and overlap of foraging habitat between species and populations that may enhance their utility as indicators of Southern Ocean ecosystems. The results highlight the importance of upwellings due to interactions of the Antarctic Circumpolar Current with large-scale bottom topography, and the corresponding localised increases in the productivity of the Polar Front ecosystem.

## Introduction

Sooty (*Puffinus griseus*) and short-tailed shearwaters (*P. tenuirostris*) are abundant seabirds that range widely across global oceans [1], breeding principally in New Zealand, south-eastern Australia, and southern South America [2], [3]. They are of cultural and economic importance for Māori and Tasmanian aboriginal societies [4], [5], important as predators within their marine ecosystems, and as engineers within their island ecosystems due to their burrowing and deposition of biological material [6], [7], [8]. Understanding the foraging ecology of these birds is important for continued monitoring of their populations [9], conservation and management of their island ecosystems [10], and facilitating their use as indicators of Southern Ocean prey stocks [9], [11].

Breeding adult sooty and short-tailed shearwaters forage locally to the colony in order to provision their chicks at a maximal rate, but periodically undertake long foraging trips to Antarctic waters [12], [13], [14], [15]. During long trips the adults forage to replenish their own body mass as well as to provision their chicks [12]. This pattern has also been reported for other species [e.g. 16]. New Zealand sooty shearwaters make long foraging trips in a spatially bimodal pattern, utilising foraging grounds around the Polar Front, to the south-east and south-west of New Zealand [1], [13]. The foraging patterns of these birds were recently examined in relation to bathymetry, sea surface temperature, primary productivity, and surface wind speeds [13]. However, questions remain regarding the foraging behaviour of these shearwaters in the high latitude regions of the Southern Ocean, including their apparent lack of use of the Polar Front zone directly south of New Zealand, and the overlap of their foraging ranges with those of conspecifics such as short-tailed shearwaters [2].

Many studies have shown that oceanic winds can influence the flight of seabirds during foraging trips [e.g. 17], [18], [19], [20], [21]. The weather systems and wind patterns south of New Zealand and Australia generally comprise polar easterlies near the Antarctic continent, and westerly winds around and to the south of the Subantarctic and Polar Fronts (∼50–55°S). North of the Subantarctic Front, alternating low- and high-pressure systems result in variable winds [22]. It seems likely that oceanic winds should have an effect on the foraging strategies of these birds, and plausible that a spatially bimodal foraging pattern might be due in part to wind considerations.

The study of wide-ranging marine predators such as these is a challenging field. Electronic tagging technologies typically play an vital role in such studies [23], but greatest insight can potentially be obtained by combining tag data with other information such as ship-based observations of predators and prey, hydrodynamic model data, and ocean parameters [11], [23], [24], [25]. Here, we integrate tracking data from sooty and short-tailed shearwaters, a long-term data set of at-sea observations of seabirds, pelagic trawl data, records of near-surface zooplankton, and remotely-sensed ocean surface wind data to provide a synoptic view of the foraging behaviour of these seabirds. Our aims were to determine the extent to which oceanic winds influenced foraging behaviour, the overlap in foraging habitat between species and populations, and to examine the relationships between foraging and potential prey distributions and oceanographic features.

## Methods

### Ethics statement

All protocols employed in the sooty shearwater tracking study were approved by the University of California at Santa Cruz Institutional Animal Care and Use Committees, the Wellington Conservancies of the Department of Conservation, New Zealand, and the Kia Mau Te Tītī Mo Ake Tōnu Atu (Keep the Tītī Forever) research programme. The South Australian short-tailed shearwater study was approved by the Department of Primary Industries and Resources of South Australia (ethics number 16/03), and the Department of Environment and Heritage (permit number A24684). The Tasmanian short-tailed shearwater study was approved by the University of Tasmania Ethics Committee (A0008138) and the Nature Conservation Branch of Tasmanian Parks and Wildlife Service (permit FA 05151). The SO-CPR survey, shipboard surveys of seabirds, and pelagic trawls were approved by the Australian Antarctic Science Advisory Committee (AAS Projects 472, 2208, and 2070), with AMLR Act permits 96/1 (trawls) and 05-10_047 (SO-CPR).

### Data collection – sooty shearwater foraging trips

Tagging was conducted on breeding adult birds on Whenua Hou (Codfish Island), New Zealand (167°39′E 46°46′S) and Mana Island, New Zealand (174°50′E 41°06′S). Details are given by Shaffer et al. [13]. The tags used were Lotek LTD 2400 archival data loggers (Lotek Wireless, St. John's, Newfoundland). These tags yielded one location fix per day, and also recorded light intensity, temperature, and pressure at a user-programmable sampling rate. The pressure data were used to infer diving activity, and the temperature data to refine position estimates by matching against sea surface temperature data [13], [26]. Eight birds were tagged for a single foraging trip with tags set to record at either 24 or 32 seconds (referred to as fast sampling rate tags). This sampling rate was chosen to provide reasonably fine-scale information on diving activity. A subsequent deployment of 19 birds was made using a slower sampling rate (432 seconds), designed to record behaviour throughout the remainder of breeding and the six month migration that followed [1]. The slower sampling rate reduces the amount of data collected each day, and so allows for longer times between recovery and download of tags.

### Data collection – short-tailed shearwater foraging trips

Tagging was conducted on breeding adult birds in two separate studies: one from two South Australian offshore islands (Althorpe Island, 136°52′E 35°22′S, and St Peter Island, 133°35′E 32°17′S) and the other from an offshore Tasmanian island (Wedge Island, 140°40′E 43°08′S). Details of the South Australian studies are given in [27]. Briefly, these birds were tagged with KiwiSat 202 satellite transmitters (Sirtrack Ltd, North Havelock, New Zealand), with a transmission interval of 60s. In this study we use data from long foraging trips, comprising a single long trip from each of 11 individual birds, spread over three breeding seasons (Feb/Mar 2005, n = 4; Feb 2006, n = 1; Feb/Mar 2007 n = 6). Due to transmitter loss and battery failure, none of these 11 trips was completely recorded. However, all but one trip were recorded to at least 50°S, and three trips were recorded to south of 60°S [27]. Thus, these records give a reasonable indication of the outward leg of long foraging trips by these birds. The Tasmanian birds were tagged with light-based geolocation tags (Lotek LTD 2400 and BAS Mk3, British Antarctic Survey). Long foraging trip data from February and March of 2006 (n = 2 birds, 5 trips total) and 2008 (n = 2 birds, 5 trips total) were used here.

### Data collection – at-sea observations of shearwaters

The at-sea observations were taken from data collected by the Australian Antarctic programme over the 1980/81 to 2005/06 austral summer seasons [28], [29]. The details of the methodologies used to collect observations and physical oceanographic and environmental data are described elsewhere [30], [31]. Briefly, observations of the numbers and behaviours of all species of seabirds present within a 300m forward quadrant of the ship were recorded continuously while the vessel was underway during daylight hours. Ship-followers were excluded from all analyses, following the BIOMASS Working Party on Bird Ecology protocols [32]. Ship-followers typically associate with the vessel for extended periods, either following the vessel at the stern or circling the vessel, or both. Neither sooty nor short-tailed shearwaters are generally regarded as ship-followers, typically flying in straight lines and only detouring around the vessel if required to avoid collision.

### Data collection – fish, squid, and zooplankton distributions

**Figure 1:**
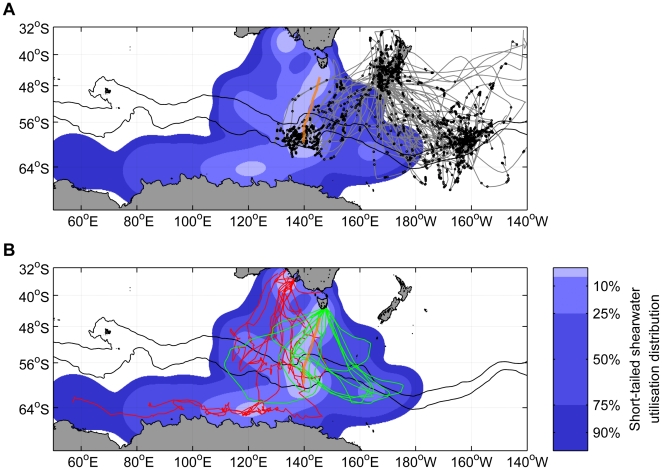
Sooty and short-tailed shearwater foraging in the Southern Ocean. A. Sooty shearwater tracks (grey lines) and dive locations (black dots), with the short-tailed shearwater habitat utilisation from panel B included for reference. Note the overlapping use of the Polar Front zone around 140°E. The northern and southern branches of the Polar Front (black) and the trawl transect (dotted orange) are shown. B. Short-tailed shearwater tracks from two South Australian islands (red lines) and from Wedge Island, Tasmania (green lines), and their corresponding combined habitat utilisation (background colours).

Mesopelagic fish and squid distributions in the Polar Front region around 140°E were obtained from an Australian voyage conducted during September 1996. Fish and squid sampling was undertaken using an International Young Gadoid Pelagic Trawl with a multiple-opening pelagic net (MIDOC). Four depth strata were sampled at each station, at nominal depths of 0–250m, 250–500m, 500–750m, and 750–1000m [33]. 37 trawls were made from 5–20 September, on a northward transect from approximately 140°E, 60°S to 146°E, 46°S (Figure 1).

Near-surface zooplankton data were obtained from the Southern Ocean Continuous Plankton Recorder (SO-CPR) survey [34]. In this survey, the CPR instrument is towed at a constant depth of ∼10m, at ∼100m behind the vessel. A 270 µm mesh is used to filter the water flowing through the instrument and the samples preserved in formalin. In subsequent laboratory analysis, the silks are cut into segments representing 5 nautical miles (referred to as “tow segments”) and all zooplankton on the silk identified [34], [35]. The SO-CPR survey has been operating since 1991, and due to the relative ease of deployment of the instrument, the survey has broad coverage of the waters south of Australia, and to a lesser extent, New Zealand. We used data from 48 voyages that surveyed areas of the Polar Front zone between 130°E and 200°E during the months of February–April. These data comprised a total of 1288 tow segments within the Polar Front zone — that is, within the northern and southern branches of the Polar Front, as defined by Sokolov & Rintoul [36]. The continuous plankton recorder survey effort in the Polar Front zone was mostly concentrated between 130°E and 155°E, with particularly heavy coverage at 140°E and 150°E resulting from repeat sampling along the World Ocean Circulation Experiment SR3 transect (∼140°E) and by the Japanese vessel *Shirase* along 150°E. We examined the pooled abundances of all copepod taxa, since copepods are a common prey of myctophids [37], [38], [39]. We note that the patterns of total zooplankton abundance were very similar to those of copepods.

### Physical oceanography

Mean positions of Southern Ocean fronts were obtained using the technique of Sokolov & Rintoul [36]. The Polar Front zone is defined here as the zone between the northern and southern branches of the Polar Front. Estimates of vertical water velocity at 1095m depth were obtained from the CSIRO Mk 3.5 model [40]. Surface chlorophyll concentrations derived from satellite ocean color measurements were based on eight day mean level 3 standard mapped images of chlorophyll on a global 9 km equidistant cylindrical grid from SeaWiFS and were obtained from the Goddard Space Flight Center [41]. Except where otherwise specified in the text, we use surface chlorophyll to refer to chlorophyll-*a* detected by satellite and use this quantity as a proxy for phytoplankton biomass.

### Analyses

All analyses were conducted in Matlab (Mathworks, Natick MA, 2009) and R (R Foundation for Statistical Computing, Vienna 2009).

At-sea observations from all years were pooled. The continuous observations were binned into records representing 10-minute surveys. There were a total of 13097 10-minute at-sea surveys during February–April in the region 60°E–200°E, 40°S to the Antarctic continent. Data for short-tailed and sooty shearwaters were pooled, as these species are difficult to separate at sea. Shearwaters were sighted in 3266 of these surveys, with an estimated total of approximately 110000 individuals. The at-sea data were collected on Australian Antarctic resupply and scientific voyages, and so the survey effort is generally concentrated to the south and west of Tasmania, extending to the Antarctic continent. There was also survey effort in between Tasmania and Macquarie Island (154°52′E, 54°37′S), but only very limited survey effort in the region south of Macquarie Island. At-sea observations of shearwaters with the behavioural category of ‘feeding’ were extremely sparse: of the 3266 surveys in which shearwaters were sighted, only 38 contained a record of feeding shearwaters. We did not examine these records in detail. To assist in visual interpretation, the spatial distribution of observed densities was smoothed by local scatterplot (Lowess) smoothing applied to log10(*x*+1)-transformed densities [42].

The processing steps applied to the sooty shearwater geolocation tag data in order to obtain daily position and diving activity estimates have been described [1], [43]. The Tasmanian short-tailed shearwater geolocation data were processed similarly, although these data were not processed to determine dive locations. The South Australian satellite-tagged short-tailed shearwater positions were filtered using the method of McConnell et al. [44].

The archival tags deployed on the sooty shearwaters allow diving behaviour to be estimated. However, the tags using slow sampling rates are unlikely to correctly represent behaviours that occur on relatively short time scales. Thus, we examined dive depths only from fast-sampling archival tags. The times of local sunrise and sunset were calculated for each dive [45]. Sooty shearwater diving activity recorded with fast sampling tags was divided into two sets, such that dives south of 50°S (i.e. associated with foraging in Antarctic waters) were in one set, and the remainder in the other. The distributions of dive times and maximum dive depths were compared between the two sets.

Diving information was not available for the short-tailed shearwaters, and so we used an estimate of habitat utilisation distribution to infer broad patterns in foraging effort. The habitat usage was computed using a kernel density estimator, with a cross-validation selection of smoothing parameter [46], [47].

**Figure 2:**
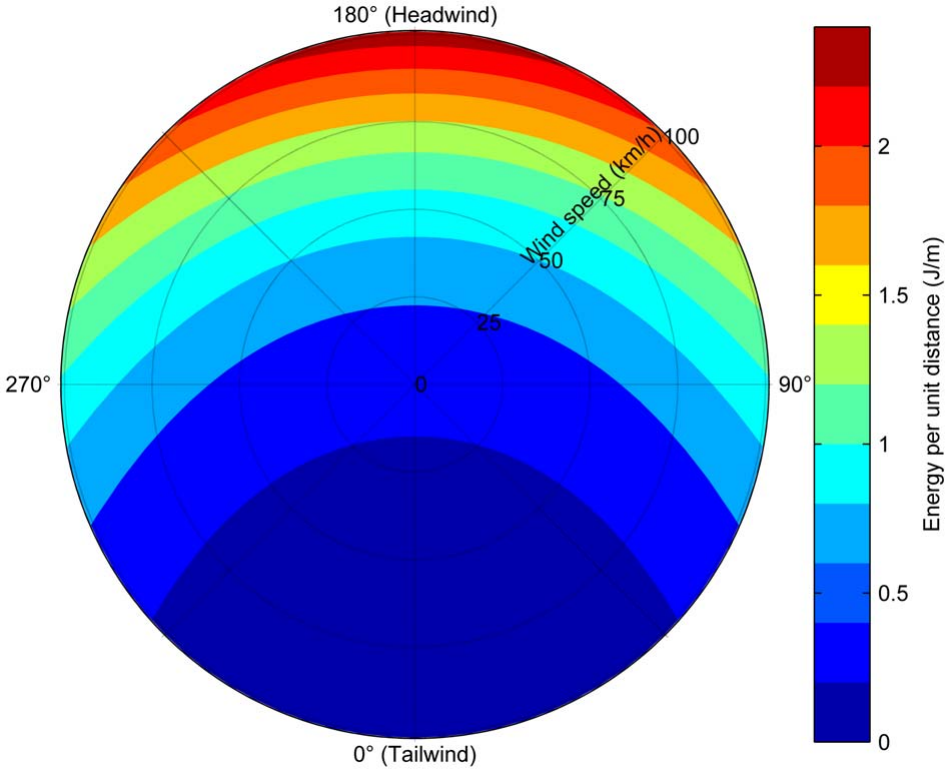
An example of the wind cost function used for the cost simulations. Costs are shown for a range of wind speeds and angles. This example is for a sooty shearwater carrying no food payload. Angles are relative to the bird flight direction: an angle of 0° corresponds to a tailwind, 90° and 270° to cross-winds, and 180° a headwind.

To gain a perspective on the overall effect of wind on potential foraging flights, grids covering the birds' potential foraging grounds were constructed, with 2.5° longitude by 1.25° latitude bins. The wind cost of visiting each grid cell was calculated using method based on that of Felicísimo et al. [48]. Each simulated foraging flight was broken into 6-hour segments. For a given simulated flight segment, the associated wind was used to calculate the energetic cost of that segment. The speed of the bird over the ground (ground speed; 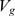) can be written as a function of the speed of the bird through the air (airspeed; 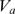) and wind speed and direction [49]:
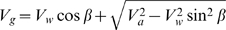
where 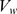 is the wind speed and 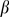 is the direction of the wind with respect to the flight track. The theoretical power required for a bird in level flapping flight is given by [50], [51]:
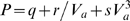
where *q*, *r*, and *s* are constants that incorporate aerodynamical flight parameters such as wing span and body mass [50]. We assumed that the birds fly at their maximum range speed (that is, the airspeed that yields maximum distance over the ground per unit energy expended). For a given wind speed and direction, 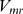, and the corresponding 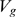 and *P*, can be found by minimising 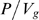 [49]. An example of the resulting cost function is shown in Figure 2, and is minimal for tailwinds, moderate for cross-winds, and heaviest for head winds.

For the flight parameters (*q*, *r*, and *s* above), we used a wing span of 1.03m and wing area of 0.0893m^2^ for sooty shearwaters and 0.93m and 0.076m^2^ for short-tailed shearwaters [52]. Sooty shearwaters embarking on a long foraging trip were assumed to have a body mass of 790g and carrying no food payload [12]. Adult birds gain body mass on long foraging trips, and on their return trips we assumed a body mass of 870g and a food payload of 193g [12]. For short-tailed shearwaters the corresponding values were 570g (outward body mass), 590g (return body mass), and 156g (return food payload) [14].

The cost of an individual simulated foraging trip was calculated as the sum of the costs of its 6-hour segments. We used near-surface wind data from the NCEP/DOE Reanalysis 2 data set (http://www.esrl.noaa.gov/psd/). These are model-based estimates of the 10m wind speed and direction, at 6-hour intervals and on a spatial grid of approximately 2° resolution. Outward flight legs were simulated as flights from the colony to the centre of the destination grid cell, and in the other direction for return flights. The intervening foraging time was not considered for cost calculations. 10000 foraging trips were simulated to each grid cell. The departure date of each simulated foraging trip was randomly sampled from the departure dates of the actual (recorded) foraging trips.

In the absence of wind effects, the energy expended during flight at constant speed is proportional to the distance travelled. Including wind effects introduces variations about this relationship. Thus, to best illustrate the spatial variability in flight costs due to wind effects, the costs are presented as residuals (percentages with respect to a smooth fit of cost against distance, fitted using natural cubic splines in the R splines library). In order to make the residuals relevant to potential Southern Ocean foraging locations, only grid points south of the colony and within 3500km (sooty shearwaters and Tasmanian short-tailed shearwaters) or 5000km (South Australian short-tailed shearwaters) of the colony were included in the fit.

## Results

### Foraging flights and spatial distributions of shearwaters

Shearwaters from all three tracking studies foraged in the vicinity of the Polar Front (Figure 1). Sooty shearwaters made use of two principal foraging areas: one to the south-west of New Zealand at approximately 140°E, and the other to the south-east at approximately 160°W (Figure 1a). Of the 31 long foraging trips made by sooty shearwaters, 21 were directed to the south-eastern foraging zone (by ten individual birds), and ten to the south-west (by four birds). Individual birds almost exclusively made long foraging trips in only one direction: only one bird made trips to both the south-western and south-eastern zones. The South Australian short-tailed shearwaters foraged from ∼110°E–150°E, with particularly high usage of the region around 140°E (Figure 1b). Foraging flights were generally made with an approximately southward outward leg followed by east-to-west foraging. The South Australian short-tailed shearwaters also foraged south of the Polar Front, as far west as 55°E in waters at or south of the southern boundary of the Antarctic Circumpolar Current (∼64°S). The foraging flights of the Tasmanian short-tailed shearwaters were consistently made in a clockwise direction, heading to the Polar Front zone at ∼165°E and then foraging east-to-west, with an approximately north-east return route to their colonies (Figure 1b).

**Figure 3:**
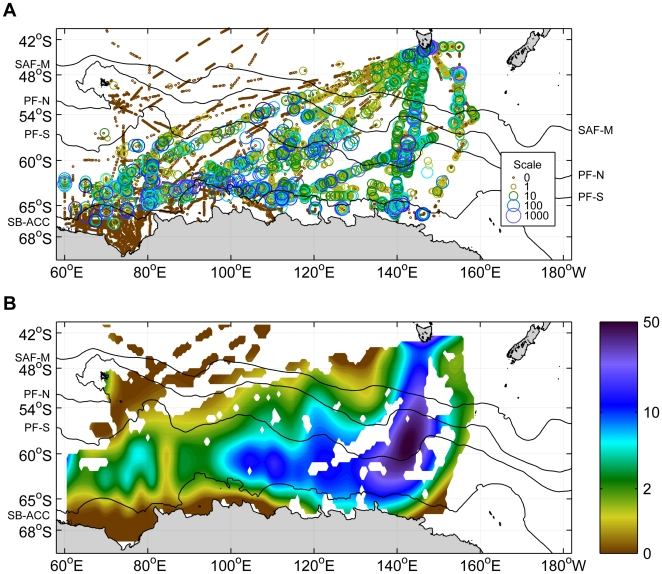
Observed at-sea densities of sooty and short-tailed shearwaters in the Southern Ocean. A. Individual survey records (number of birds per 10-minute survey). B. Smoothed density surface fit with local scatterplot smoothing (see text). Locations of Southern Ocean fronts from north to south are shown in black: SAF-M, middle branch of the Subantarctic Front; PF-N, northern branch of the Polar Front; PF-S, southern branch of the Polar Front; SB-ACC, southern boundary of the Antarctic Circumpolar Current.

At-sea observations of shearwaters were generally distributed over offshore waters around and to the south of the Polar Front, from approximately 60°E to 150°E (Figure 3). The highest densities were observed in the vicinity of the Polar Front around approximately 140°E, and also close to the coast of Tasmania. The high densities of at-sea observations of shearwaters in the Polar Front zone at ∼140°E were reasonably consistent across years. Six voyages intersected this zone (1993, 1994, 1998, 2000 (2), and 2006). On four of these voyages the observed densities of shearwaters were higher in this zone than the remainder of the voyage (Wilcoxon rank sum test, *p*<0.001 in each case). In the remaining two voyages the densities were not significantly different in this zone compared to the remainder of the voyage.

### Diving behaviour of sooty shearwaters

**Figure 4:**
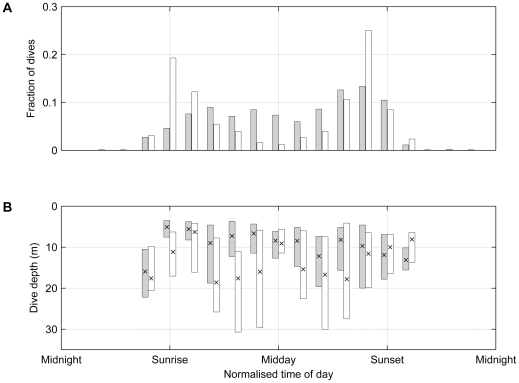
Diel distributions of sooty shearwater diving activity. A. Distributions of sooty shearwater dives with respect to time of day. B. Dive depths with respect to time of day. The median (cross) and interquartile range (bars) are shown. Grey bars indicate dives made from 30°S–50°S, and white bars indicate dives made south of 50°S.

Sooty shearwater dives recorded by fast sampling archival tags north of 50°S (n = 801 dives by seven individual birds) occurred from just before sunrise through to sunset, with broad peaks of dive activity centred on mid-morning and late afternoon (grey bars in Figure 4a). Dive activity south of 50°S (n = 933 dives by two individual birds) also occurred throughout the day, but showed distinct peaks just after sunrise and just before sunset (white bars in Figure 4a). The diel distributions of dive activity north and south of 50°S were significantly different (p<0.001, χ^2^_12_ = 245.1).

The median maximum dive depth (fast tags; all dives combined, n = 1734) was 10.1m (interquartile range IQR 12.8m). The dives south of 50°S (median maximum depth 11.9m, IQR 14.5m) were deeper than those north of 50°S (8.5m, IQR 10.2 m; Wilcoxon p<0.001). The dive depths tended to be shallowest around sunrise and sunset, becoming deeper during mid-morning and mid-afternoon, and shallow again around midday (Figure 4b).

### Simulations of wind costs during long foraging flights

**Figure 5:**
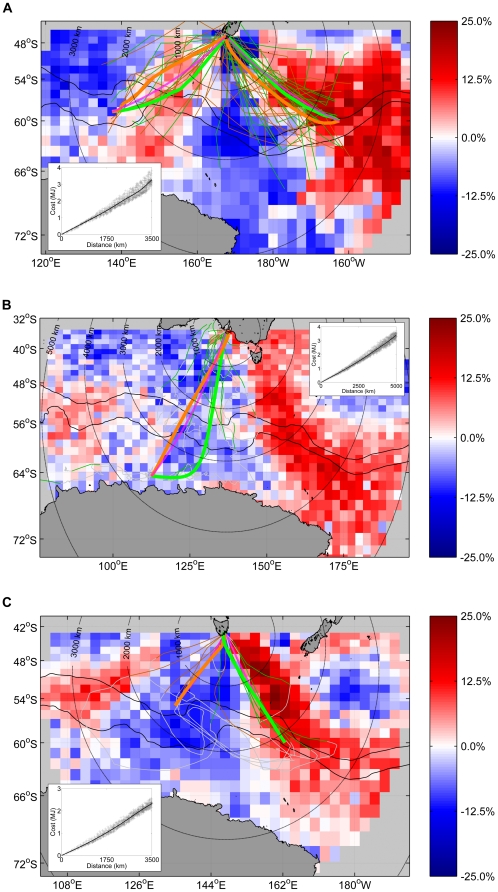
Simulated wind costs of long foraging trips by sooty and short-tailed shearwaters. A. Simulated wind costs (background colours) for sooty shearwaters. B. For South Australian short-tailed shearwaters. C. For Tasmanian short-tailed shearwaters. Costs are shown as percentage residuals from smooth regression of cost against distance. For example, a value of 25% indicates that the cost to visit the area in question is 25% higher than the average cost for potential foraging locations at the same geographical distance from the colony. Insets show regressions of cost against distance. The thin green lines show outward flights from the colony; the thick green lines show the simulated minimum-cost paths from the colony to representative points on the birds' foraging grounds. The orange lines show the same information for the return trips. Grey lines show the foraging components of flights (not shown in panel A for clarity). The purple lines show the direct (geodesic) routes. The northern and southern branches of the Polar Front are also shown (black).

The simulated wind costs for the three tracking studies showed similar broad patterns, with low-cost regions directly south of the colonies, and higher-cost regions extending in corridors roughly south-east and south-west from the colonies (Figure 5). The Polar Front region directly south of New Zealand, sparsely utilised by the sooty shearwaters, was both closer to the colonies and more energetically favourable (i.e. lower cost) than their observed foraging zones to the south-east and south-west of New Zealand.

The observed South Australian short-tailed shearwater foraging activity corresponded to energetically-favourable areas (Figure 5b), whereas both the Tasmanian short-tailed shearwaters (Figure 5c) and the sooty shearwaters (Figure 5a) made outward flights to the south-east of their colonies, into apparently energetically-costly areas. Although variable, the prevailing wind over much of the Southern Ocean south of Australia and New Zealand was approximately north-westerly (i.e. blowing towards the south-east). South-easterly flight from the colonies was therefore accompanied by favourable (near-tailwinds) on the outward leg, but entailed return flights against headwinds. Because the wind cost function is asymmetric (that is, the penalties associated with head winds are proportionally higher than the benefits provided by tailwinds), such trips were more energetically costly overall.

For short-tailed shearwaters the minimum-cost flight paths from the simulations generally showed good agreement with the actual flight paths recorded from the tags. The simulated minimum-cost flight paths for South Australian short-tailed shearwaters foraging in regions south of the Polar Front zone involved a roughly southerly flight to near-Antarctic waters, followed by westerly flight using the polar easterly winds near the Antarctic coast as tailwinds (thick green line in Figure 5b), in good agreement with the observed flight paths of the tagged birds. These birds did not tend to forage to the east of their colonies south of the Polar Front, possibly because this would entail return flights against unfavourable winds. The minimum-cost return flight path from Antarctic waters was close to the most direct route (purple line in Figure 5b). Although none of the tags survived long enough to capture a return flight path, the at-sea densities (Figure 3) are consistent with reasonably direct return flights, as are previously published tracks from short-tailed shearwaters [53].

### Physical oceanography of the foraging zones

**Figure 6:**
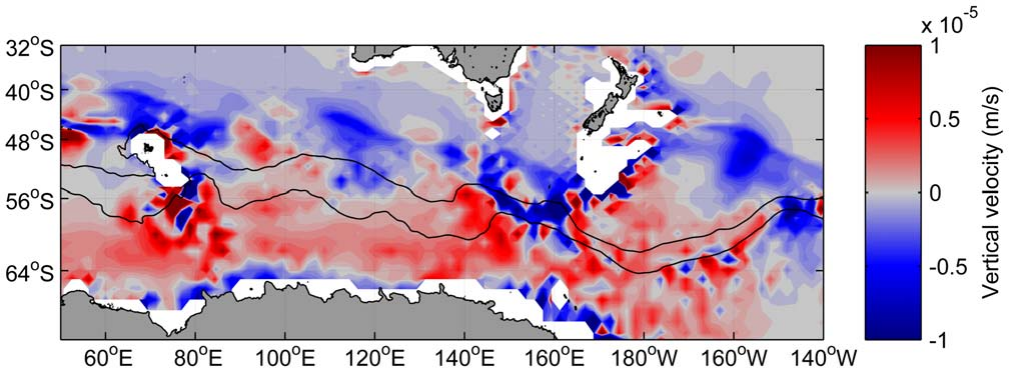
Vertical water velocity at 1095m in the Southern Ocean. Twenty-year mean vertical velocity from the CSIRO Mk3.5 climate system model. The northern and southern branches of the Polar Front are shown.

**Figure 7:**
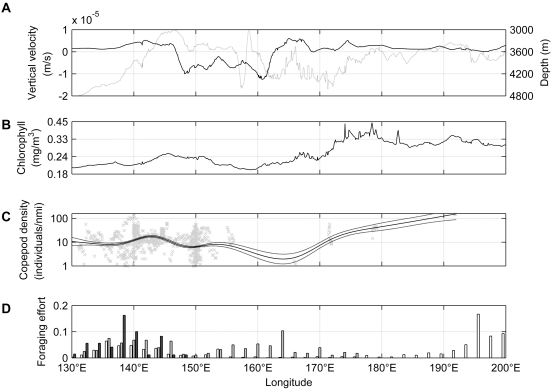
Shearwater foraging in the Polar Frontal zone, in relation to oceanographic processes and copepod abundances. A. Longitudinal distributions in the Polar Frontal zone of water vertical velocity at 1095m depth (solid line, positive = upward) and water depth (dotted). B. Mean summer surface chlorophyll-a from SeaWiFS satellite estimates. C. Near-surface copepod density from continuous plankton recorder surveys (points = individual tow segments, black lines = mean±SE fit by generalised additive model). D. Shearwater relative foraging effort within 3° of latitude of the Polar Frontal zone (white = sooty shearwaters, black = South Australian short-tailed shearwaters, grey = Tasmanian short-tailed shearwaters).

Strong, vertically-coherent, topographically-driven upwelling is present in several sectors of the Polar Front zone in the region of interest, notably around 140°E and 165°E, and east of 170°E (Figures 6 and 7a). These zones of strong upwelling extend over several hundred kilometres and are driven by the interaction of the eastward-flowing Antarctic Circumpolar Current with large-scale bathymetric features: the South-East Indian Ridge (140°E), and the Pacific-Antarctic Ridge (east of 165°E). These regions of upwelling correspond to the regions of high surface chlorophyll concentrations (Figure 7b), and with shearwater foraging effort in the Polar Front zone (Figure 7d).

Productivity is generally higher south of the Polar Front, due to a range of factors including higher upwelling rates, iron input from melting sea ice, and shallower mixed layers [54], [55]. The at-sea observations and the tracks of the South Australian short-tailed shearwaters extend into this zone, and the observed at-sea shearwater densities (Figure 3) tend to coincide with the changes in the upwelling rates and primary production (e.g. the higher densities of shearwaters observed west of approximately 85°E correspond to the region of strong upwelling and intensive chlorophyll bloom near the southern Kerguelen Plateau [54]).

### Potential prey abundances

**Figure 8:**
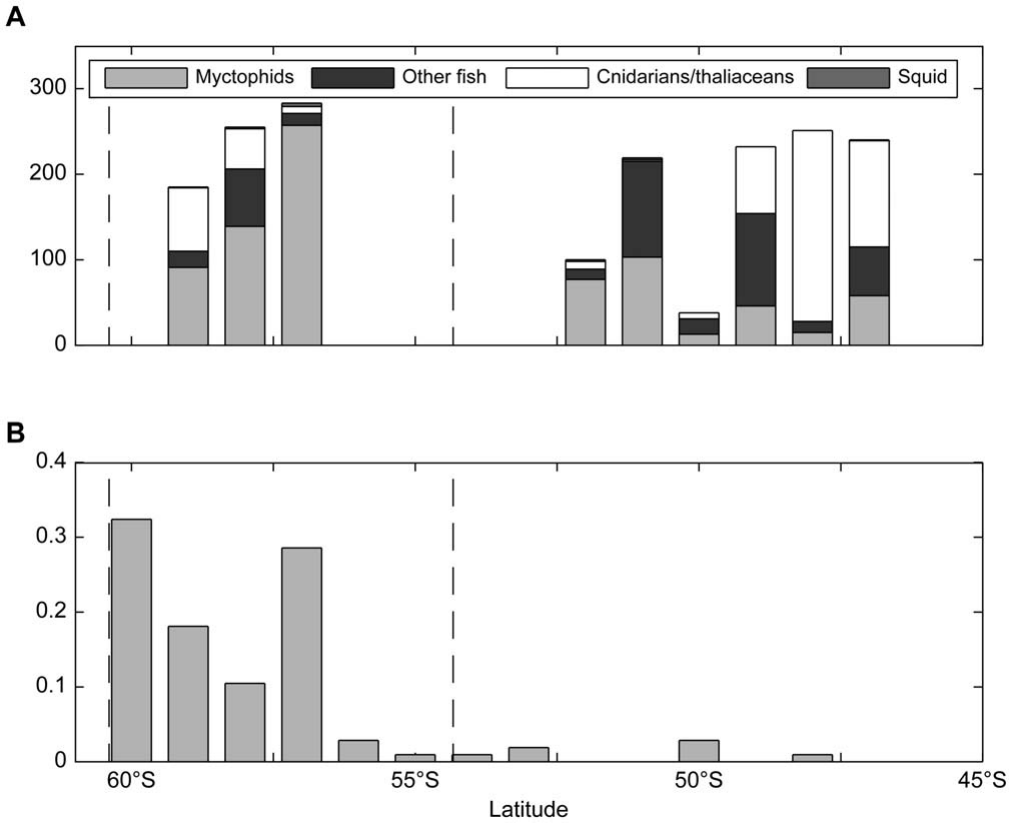
Latitudinal distributions of pelagic trawl abundances and sooty shearwater diving activity. A. Pelagic prey abundances (individuals/trawl) in the 60°S to 45°S latitude band, 0–500m depth, at approximately 140°E. B. Sooty shearwater relative dive distribution in the 135°E–145°E sector for the same latitude range. Dotted lines show the latitudes of the southern and northern branches of the Polar Front.

The trawl transect was made in the longitude band 140°E–146°E, and gives an indication of latitudinal variation in pelagic prey availability. North of 50°S, the trawls from the top 500m of the water column were dominated by pyrosomes (65% by number) and salps (11%), and the fish *Photichthys argenteus* (8%; Figure 8). From 50°S–54°S, myctophids overwhelmingly dominated (90%), but these numbers were strongly skewed by one particularly large haul of *Electrona carlsbergi*, which contributed 61% of the total myctophids caught in this latitude band. Temporarily excluding this haul of *E. carlsbergi* (Figure 8 is presented with this haul of *E. carlsbergi* excluded), the total myctophid component in the 54°S–50°S band was 54% (mainly *Gymnoscopelus microlampas*, 15%; *G. piabilis*, 8%; and unidentifiable *Protomyctophum*, 8%). Other fish (41%; mainly unidentifiable fish, 17%; and *Persparsia kopua*, 13%) and small numbers of cnidarians and salps (3%) made up the remainder. South of 54°S the hauls were again dominated by myctophids (67%; mainly *G. braueri*, 22%; *Krefftichthys anderssoni*, 15%; and *E. antarctica*, 9%), with smaller numbers of other fish (14%; mainly *Bathylagus* species, 6%; and unidentifiable fish, 4%), and the coronate scyphomedusae *Atolla wyvillei* (11%) and *Periphylla periphylla* (6%).

Figure 7c shows the longitudinal distribution of near-surface copepod abundances in the Polar Front zone. Abundances were highest around 145°E and to the east of 170°E, although survey effort in the latter sector was sparse. The regions of higher copepod abundance generally coincided with the regions of persistent upwelling (Figure 7a), high surface chlorophyll concentrations (Figure 7b), and with shearwater foraging effort (Figure 7d).

## Discussion

### Likely prey of shearwaters on long foraging trips

The diet of short-tailed shearwaters has been relatively well described [14], [56], [57], and on long foraging trips they predominantly consume myctophids (lantern fish), with a small euphausiid component (likely *Euphausia vallentini*) [56], [57]. Some myctophids, including the relatively common *E. antarctica*, *G. braueri* and *K. anderssoni* are rich in wax esters [58], [59], which act as long-term energy reserve molecules rather than immediate energy supply [60] and they form an important component of stomach oil that is fed to chicks [61]. A number of studies show that sooty shearwaters consume a wide range of crustaceans, fish, cephalopods, and salps [e.g. 62], [63], [64], [65]. The specific prey of sooty shearwaters on long foraging trips has not to our knowledge been studied, although it has been suggested that in Antarctic waters sooty shearwaters also principally forage for myctophids [12]. The results from our study support this suggestion.

The observed foraging zones of the sooty shearwaters in this study, like those of the short-tailed shearwaters, are consistent with the general patterns of myctophid distribution in the Southern Ocean. Myctophids are widely distributed and abundant, particularly from the Polar Front zone south to the Antarctic continental shelf [37], [38], [66]. Around and to the south of the Polar Front zone, myctophids are commonly recorded within 200m of the surface [66], [67], and rise to the surface waters at night [68], [69]. Some myctophid species are also found north of the Polar Front zone (e.g. *E. carlsbergi*, *G. microlampas*, *G. piabilis*), but inhabit deeper waters (250–600m) in these areas [67], [70], which may limit their availability to surface-diving seabirds. These general distribution patterns were observed in the trawl data, with increased myctophid densities south of 50°S.

The observed timing of sooty shearwater dive activity in Antarctic waters is also consistent with the proposition that the sooty shearwaters were foraging for myctophids, or other prey that migrate to near-surface waters during night-time hours. Sooty shearwaters use visual prey detection methods, scanning for underwater prey by immersing their heads before diving [71], [72]. The observed distribution of dive activity in Polar Front waters (peaking just after sunrise and just before sunset, with no night-time diving) might represent a compromise between increased prey availability (proximity to surface) and decreased visual prey detectability during the hours of darkness. Sooty shearwaters have been observed to feed on myctophids during the day in the presence of other marine predators that drive the fish towards the surface waters [65], and shearwaters are commonly observed in association with cetaceans in Antarctic waters [e.g. 73]. The dive depths reported here for sooty shearwaters are underestimates of the true maximum dive depths, due to the low sampling rate (one sample per 24 or 32s) of the tags relative to the time spent by the birds at the deepest part of their dives. Capillary tube measurements show that sooty shearwaters are capable of diving to depths exceeding 60m [74], [75].

The relatively low sampling rate of the tags also means that the probability of failing to detect a dive is greater for dives of shorter duration. It is possible that our finding of dawn and dusk diving in high latitudes has been confounded by this bias. If, for example, dive activity was in reality constant throughout the day, but the dive durations were shorter around midday and longer around dawn and dusk, then the distribution of detected dives would peak around dawn and dusk, as we found here. However, we compared the diel distribution of dive activity from fast sampling tags (sampling rate 24s or 32s) to the distribution obtained from slow sampling tags (432s; n = 3301 dives total by 18 individual birds; results not shown), and found the distributions to be very similar. Missed detections of short duration dives should be more pronounced with the slow sampling tags, and since the distributions match, we do not believe that the diel distribution of dive activity is an artefact of sampling bias in this case.

Cherel et al. [57] reported that *Euphausia vallentini* were the main crustacean prey from long-trip samples in short-tailed shearwaters from Tasmanian (Bruny Island) colonies. *E. vallentini* is distributed to the north of the Polar Front [14], [76], [77]. The short-tailed shearwaters in this study, particularly the South Australian birds, were observed to forage in this region. Although *E. vallentini* have been reported in sooty shearwater diet samples [63], it seems unlikely that sooty shearwaters principally target this species on long foraging trips, since their primary foraging zones lie around and to the south of the Polar Front zone (Figure 1). The South Australian short-tailed shearwaters also foraged well south of the Polar Front, in waters where *E. superba* (Antarctic krill) is the most abundant euphausiid. Birds tracked from Montague Island, NSW [53] were observed to travel to Antarctic waters where krill is common, as did a post-breeding bird from French Island, Victoria [78]. Connan *et al.* [56], [79] reported that lipid concentrations from 14 short-tailed shearwaters after long foraging trips did not match those of *E. superba*, and concluded that this euphausiid might not be a major component of their diet. This result appeared to contradict the above tracking studies as well as earlier stomach samples and observed predation of shearwaters on *E. superba* [80], [81]. However, the results of Connan *et al.* [56], [79] were obtained from birds breeding on Bruny Island, Tasmania. The small sample of Tasmanian (Wedge Island) birds tracked in this study did not forage in waters where *E. superba* might be expected. Thus, this apparent contradiction might be a result of site-specific differences in foraging area preferences of birds from different regions and colonies. *E. superba* might still be an important dietary component for some populations of short-tailed shearwaters, such as those from South Australian islands.

### Wind cost analyses

One of the objectives for the current study was to evaluate whether the longitudinal distribution of sooty shearwater foraging effort in the Polar Front zone was related to patterns of oceanic winds. There is little in our results to suggest that the region directly south of New Zealand was sparsely utilized by the birds because of wind cost factors. However, wind patterns do appear to explain other broad-scale patterns in foraging, such as the lack of use of near-Antarctic waters east of about 150°E by South Australian short-tailed shearwaters. This use of wind on long foraging trips has previously been noted [e.g. 53] but not formally investigated.

Both the sooty shearwaters and the Tasmanian short-tailed shearwaters made long foraging trips to the south-east of their colonies, into apparently energetically-costly areas. Shaffer et al. [13] noted the bias of the sooty shearwaters toward the south-eastern sector, and provided several possible explanations, including potentially higher prey abundances in that zone. Our wind cost analyses suggest two further factors. First, while such trips were costly overall, the outward flight legs were generally accompanied by favourable tail and hind-quarter winds. Long foraging trips are generally initiated when the adults have poor body condition [12], [14] and so a foraging trip with minimal initial energy cost might be preferable to one that has a lower total cost (i.e. when the return leg also considered). Second, shearwaters breeding in south-eastern Australia do not appear to forage in this zone, possibly because of the high energy costs that they would incur in doing so. The south-eastern zone might therefore represent an area of reduced competition for the sooty shearwaters.

There are a number of potential concerns with the wind cost analyses. Shearwaters generally fly close to the water, where wind speeds will be lower than the estimates provided by the NCEP2 data (which are for winds at 10m above the ocean surface, where shearwaters rarely fly). Flight close to the sea surface will reduce the induced drag and therefore the energy cost of flight [82], but perhaps more importantly, means that ocean waves become a potentially important consideration. Shearwaters are known to use wave troughs and wind gradients during flight [e.g. 83], [84]. Dynamic flight techniques that exploit the interaction of winds and waves, such as gust soaring [50], [51], have the potential to dramatically reduce the energy expenditure of seabirds in flight over the open ocean. The cost function used here does not account for such techniques. The decision not to incorporate these factors into the wind cost analyses was in part a reflection of the lack of fine-scale wind and wave data over the regions of interest (which would be required to model dynamic flight costs), and also the relatively coarse spatial and temporal resolution of the seabird locations (particularly those from the geolocation tags, which provide only one position fix per day and with relatively large spatial uncertainty). The wind cost analyses must therefore be interpreted in the context of relatively large-scale behaviours (of the order of days, or hundreds of kilometres).

Further caution should be exercised in interpreting the insights into adult foraging behaviour obtained from tagged birds, since it is known that the handling of birds and presence of tags can reduce colony attendance and provisioning of chicks by the adult birds [43], [85], [ but see also 86].

### Relationship with physical oceanography in the Polar Front zone

The foraging effort of the shearwaters within the Polar Front zone was not uniform in longitude, but was focused on areas of persistent upwelling. The association of shearwater foraging activity with these areas reflects elevated prey availability (as measured by near-surface copepod abundances), resulting either from elevated production in these areas, and/or the aggregation of prey in near-surface waters by upwelling and convergence [e.g. 87].

The persistent upwellings in the Polar Front zone are largely coincident with the surface productivity, as measured by satellite estimates of surface chlorophyll (Figure 7; see also Figure 8 in [54]). In the open ocean away from shallow regions, blooms along Southern Ocean fronts are generally initiated by upwellings due to the interactions of the Antarctic Circumpolar Current with large-scale bottom topography [54]. A large and intense topography-induced upwelling is found where the Polar Front interacts with Pacific-Antarctic Ridge, from ∼170°E–200°E, and this zone corresponds to the strongest chlorophyll bloom found in the Pacific sector of the Southern Ocean [54], and also to the sooty shearwaters' foraging zone south-east of New Zealand. The upwellings in the vicinity of the South-East Indian and Macquarie Ridges (140°E and 165°E; also foraging zones) have relatively weak signatures in surface chlorophyll distribution (Figure 7a). However, in-situ observations along the World Ocean Circulation Experiment SR3 section (140°E line) show a persistent subsurface chlorophyll maximum at depths of 50–100m [88], [89]. This subsurface maximum does not extend northward into the subantarctic zone. Parslow et al. [88] concluded that vertical processes (rather than horizontal advection) were the dominant controllers of this feature, consistent with the vertical velocity distributions shown here (Figure 6).

### Consistency and overlap of foraging areas

The overlap of foraging zones between the two shearwater species, and with commercial fisheries, is an issue of ongoing interest [2]. The Polar Front region around 140°E was utilised by shearwaters from all three tracking studies incorporated here. The range of years covered by those studies, combined with the consistency of high at-sea densities in this region, strongly suggests that this region is used repeatedly across years. This is also consistent with previously published tracks of short-tailed shearwaters from south-eastern Australia [53], [78], and with land-based observations of southward-, but not northward-flying shearwaters at Macquarie Island [90]. The south-eastern sooty shearwater foraging zone matches that recorded in 2000 (Figure 1 in [2]), suggestive of inter-annual consistency in the use of other foraging locations. The sooty shearwaters also showed foraging zone fidelity at an individual level, with only one bird foraging in both the south-eastern and south-western zones. Fidelity to mesoscale features such as fronts and upwelling zones is extremely common in pelagic seabirds [91]. Central-place foragers, such as breeding seabirds, tend to repeatedly utilise foraging zones where prey are consistent [92], [93]. The strategy of returning to learned foraging zones provides a means of counteracting the natural patchiness of marine prey [94].

The lack of foraging activity by the sooty shearwaters in the Polar Front zone sector from ∼145°E–180°E does not appear to be a result of wind-cost factors, but likely prey availability and other factors. This sector encompasses a sector of persistent downwelling (∼148°E–162°E, Figure 6). While the sooty shearwaters did not utilise the region around 165°E, this is a region of upwelling and was utilised by the Tasmanian short-tailed shearwaters. The lack of use of this particular area by the sooty shearwaters might therefore reflect differences in preferred (learned) foraging areas of the birds.

Tracking studies on other species have shown foraging distributions in similar areas to the shearwaters. Southern elephant seals *Mirounga leonina* from Macquarie Island, like the sooty shearwaters, showed a bimodal south-east/south-west foraging pattern (Figure 1b in [95]), although these seals foraged farther south than did the sooty shearwaters. Grey-headed albatrosses *Thalassarche chrysostoma* from Macquarie Island foraged just west of the sooty shearwaters' south-western foraging zone, around 180°E–190°E (Figure 5 in [96]), as did grey-headed and black-browed *T. melanophrys* albatrosses from Campbell Island [97]. Light-mantled sooty albatrosses *Phoebetria palpebrata*, also from Macquarie Island, foraged just south of the south-western zone (approximately 130–140°E, 64°S) [98]. King penguins *Aptenodytes patagonicus* from Macquarie Island foraged east and south of the island [99], including the Polar Front zone at ∼165°E where the Tasmanian short-tailed shearwaters foraged. Stomach samples from these penguins consisted of fish (primarily the myctophids *K. anderssoni* and *E. carlsbergi*) and squid (*Moroteuthis ingens* and *Mastigoteuthis* sp.). *M. ingens* is a predator of myctophids, particularly *K. anderssoni* [100]. The dives made by these penguins in the Polar Front zone were similar in pattern to those of the sooty shearwaters reported here, occurring from sunrise until mid-morning, and again from mid-afternoon until sunset, with little dive activity around midday (B. Wienecke, unpublished). The dive depths were also similar: shallowest at sunrise and sunset, increasing in depth closer to mid-day; consistent with the diurnal vertical migration of myctophids and their predators. The penguins' deepest dives (up to 170m) were well beyond the maximum diving depths of shearwaters, suggesting that such prey would be accessible to the shearwaters for a much shorter period of each day. However, they can cover a much greater distance over this shorter time period.

Upper-level trophic predators, including seabirds, are commonly used as indicators for ecosystem monitoring [e.g. 101], [102], [103] and fish stock assessment [e.g. 104]. Clearly, their value in this role is determined by the extent to which variations in indices derived from these predators reflect changes occurring at lower trophic levels of the ecosystem [e.g. 105]. The use of sooty and short-tailed shearwaters as indicators of Southern Ocean ecosystems is compromised to some extent by their annual migrations and corresponding exposure to factors external to the Southern Ocean [e.g. 106], [107]. However, our results suggest that during the breeding season, these birds are consistent in their foraging habitats. The foraging overlap between the two species, and between populations, and the similarities in their foraging areas to those of other marine predators, suggests that these birds might be useful as indicators of aspects of the Southern Ocean ecosystem, particularly of the Polar Front zone. Combining indices from multiple colonies and/or different species might allow variations in breeding success due to localised factors (e.g. density dependence, or predation from introduced mammals) to be disentangled from those due to changes in the ecosystem of the Polar Front zone. Combining indicator information from populations at multiple sites, and from multiple species, can assist in reducing single-site effects, and potentially make indicators based on seabird breeding performance more robust [108]. An index of the abundance of Australian krill (*Nyctiphanes australis*) based on a combination of indices of the growth of short-tailed shearwater chicks in Tasmania and mortality occurrences in Japan has previously been proposed [109].

### Summary and conclusions

Electronic tagging technologies can provide detailed insights into the foraging and migratory behaviours of wide-ranging marine predators. In this study we combined tracking data from two shearwater species that originate from colonies separated by thousands of kilometres in New Zealand and Australia, along with ship-based and remote-sensed information to evaluate and describe the predators' foraging behaviour within the wider context of their marine ecosystem.

The birds displayed foraging site fidelities at individual and population levels, with overlap in foraging habitat between species and populations. Wind cost modelling yielded new information about the putative mechanisms that influence foraging destination. The distributions of foraging effort at broad spatial scales were consistent with patterns in oceanic winds. Prey availability modulated these patterns, and influenced foraging effort at smaller spatial scales. The spatial distribution of shearwater foraging effort in the Polar Front zone was matched by patterns in large-scale upwelling, primary production, and abundances of copepods and myctophid fish. These results offer great promise for modelling and projecting possible changes in foraging behaviour and distribution related to environmental factors.
